# Doppler ultrasound for diagnosis of soft tissue sarcoma: efficacy of ultrasound-based screening score

**DOI:** 10.1515/raon-2015-0011

**Published:** 2015-03-25

**Authors:** Satoshi Nagano, Yuhei Yahiro, Masahiro Yokouchi, Takao Setoguchi, Yasuhiro Ishidou, Hiromi Sasaki, Hirofumi Shimada, Ichiro Kawamura, Setsuro Komiya

**Affiliations:** 1 Department of Orthopaedic Surgery, Graduate School of Medical and Dental Sciences, Kagoshima University, Kagoshima, Japan; 2 The Near-Future Locomotor Organ Medicine Creation Course (Kusunoki Kai), Graduate School of Medical and Dental Sciences, Kagoshima University, Kagoshima, Japan; 3 Department of Medical Joint Materials, Graduate School of Medical and Dental Sciences, Kagoshima University, Kagoshima, Japan

**Keywords:** Doppler ultrasound, soft-part tumours, differential diagnosis, ultrasound-based sarcoma screening score

## Abstract

**Background.:**

The utility of ultrasound imaging in the screening of soft-part tumours (SPTs) has been reported. We classified SPTs according to their blood flow pattern on Doppler ultrasound and re-evaluated the efficacy of this imaging modality as a screening method. Additionally, we combined Doppler ultrasound with several values to improve the diagnostic efficacy and to establish a new diagnostic tool.

**Patients and methods.:**

This study included 189 cases of pathologically confirmed SPTs (122 cases of benign disease including SPTs and tumour-like lesions and 67 cases of malignant SPTs). Ultrasound imaging included evaluation of vascularity by colour Doppler. We established a scoring system to more effectively differentiate malignant from benign SPTs (ultrasound-based sarcoma screening [USS] score).

**Results.:**

The mean scores in the benign and malignant groups were 1.47 ± 0.93 and 3.42 ± 1.30, respectively. Patients with malignant masses showed significantly higher USS scores than did those with benign masses (p < 1 × 10^−10^). The area under the curve was 0.88 by receiver operating characteristic (ROC) analysis. Based on the cut-off value (3 points) calculated by ROC curve analysis, the sensitivity and specificity for a diagnosis of malignant SPT was 85.1% and 86.9%, respectively.

**Conclusions.:**

Assessment of vascularity by Doppler ultrasound alone is insufficient for differentiation between benign and malignant SPTs. Preoperative diagnosis of most SPTs is possible by combining our USS score with characteristic clinical and magnetic resonance imaging findings.

## Introduction

The diagnosis of soft-part tumours (SPTs) in orthopaedic primary care is not easy because of the rarity of the disease, few characteristic findings, and lack of simple diagnostic tools. Ultrasonography is a noninvasive imaging tool that is gaining popularity in orthopaedic clinics. It has already been used in daily clinics for evaluation of muscle injury^1^, rotator cuff tears^2^, entrapment neuropathy^3^, and many other conditions.^4^ The utility of ultrasound imaging in the screening of SPTs has been discussed in the past by several authors with some modifications.^5^–^9^ To improve the diagnostic accuracy of malignant tumours, more defined and complex methods of ultrasound imaging for SPTs have been reported. For instance, contrast-enhanced ultrasound is reportedly useful for differentiation between malignant and benign SPTs by analysis of contrast-enhancement kinetics^10^ or in combination with three-dimensional power Doppler.^11^ On the other hand, Chiou *et al.* used a computer-aided diagnosis (CAD) system to improve the diagnosis of malignant SPT.^12^ They analysed five features (namely area, boundary transition ratio, circularity, high-intensity spots, and uniformity) with the CAD system and achieved a sensitivity of 88.2% and specificity of 87.5%. Although this new method might be more accurate, it is not feasible for outpatient screening. An accelerated diagnostic process and appropriate treatment of patients with SPTs could be expected with the use of a simple, easy screening method using ultrasound without the requirement for a special technique. Ultrasound may also reduce the performance of unnecessary imaging studies, thus decreasing medical care costs.

Previous reports on diagnostic ultrasound imaging of SPTs describe several methods with which to judge malignancy according to the amount of blood flow seen on Doppler ultrasonography.^8^,^13^,^14^ Giovagnorio *et al*.^13^ classified the blood flow patterns of 51 superficial SPTs into 4 types. By defining hypervascular type III and IV blood flow as malignant criteria, they reported a sensitivity and specificity of 90% and 100%, respectively. Their method is recognised as one of the most useful screening techniques for SPTs.^15^

In the present study, we classified the blood flow pattern of SPTs according to the Doppler ultrasound findings and re-evaluated these previously described results.^13^ We also sought to establish new diagnostic criteria, and thus improve the diagnostic efficacy by combining Doppler ultrasound findings with other factors.

## Patients and methods

This study included 189 patients with SPTs who underwent surgery in our department and obtained a pathological diagnosis. Ultrasound imaging studies were performed at the initial visit to the orthopaedic outpatient clinic. The patients comprised 87 men and 102 women with an average age of 54 years. In total, 122 patients had benign diseases including SPTs and tumour-like lesions, while 67 patients had malignant SPTs (Table 1). This study was conducted according to the principles of the Declaration of Helsinki and approved by the institutional ethical committee.

A musculoskeletal oncologist performed the ultrasonography examinations using a 12L-RS linear-type probe of 5.0 to 13.0 MHz (Logic e series; General Electric, Fairfield, CT). According to a report by Giovagnorio *et al.*^13^, we classified the intratumoural blood flow patterns shown on colour Doppler into four groups: avascular (type I), hypovascular with a single vascular pole (type II), hyper-vascular with multiple peripheral poles (type III), and hypervascular with internal vessels (type IV).

We also established a scoring system to improve the sensitivity of differential diagnosis of benign and malignant SPTs (ultrasound-based sarcoma screening [USS] score). In addition to three ultrasound findings (echoic intensity, uniformity of internal structure, and Doppler blood flow classification), the tumour diameter was included in the USS score (Table 2). We evaluated all cases using the USS score and analysed its utility in the differential diagnosis of SPTs.

A p value of < 0.05 was considered to indicate a statistically significant difference between two groups using the chi-squared test or Student’s t test. Additionally, we established the cut-off value by receiver operating characteristic (ROC) curve analysis (Microsoft Excel; Microsoft Corporation, Redmond, WA).

## Results

**FIGURE 1.**
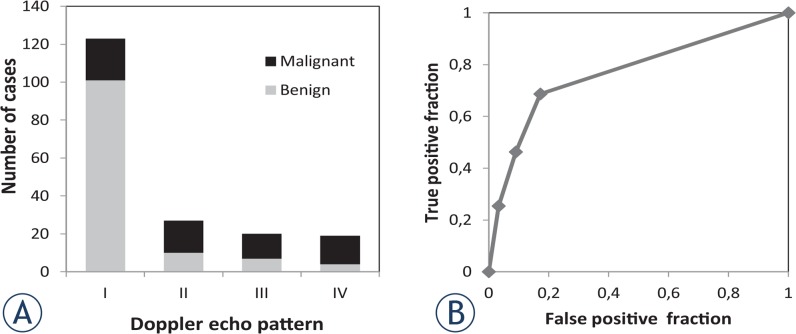
Validation of categorisation by Doppler ultrasound pattern. **(A)** All cases were categorised by their vascular pattern using Doppler ultrasound according to the classification described by Giovagnorio *et al*. The type I group included 101 benign tumours and 22 malignant tumours. In contrast, type IV comprised 4 and 15 benign and malignant tumours, respectively. **(B)** Receiver-operating curve analysis of the Doppler ultrasound classification described by Giovagnorio *et al*. The efficacy of the type III and IV pattern for diagnosis of malignant tumours was evaluated. The area under the curve was 0.77.

The rates of malignant tumours were 18% among type I, 63% among type II, 65% among type III, and 79% among type IV using the classification described by Giovagnorio *et al.*^13^ (Figure 1A). Using type III and IV vascularity as markers of malignancy, the sensitivity and specificity were 41.8% and 91.0%, respectively. ROC analysis demonstrated that the area under the curve was 0.77 (Figure 1B).

**FIGURE 2.**
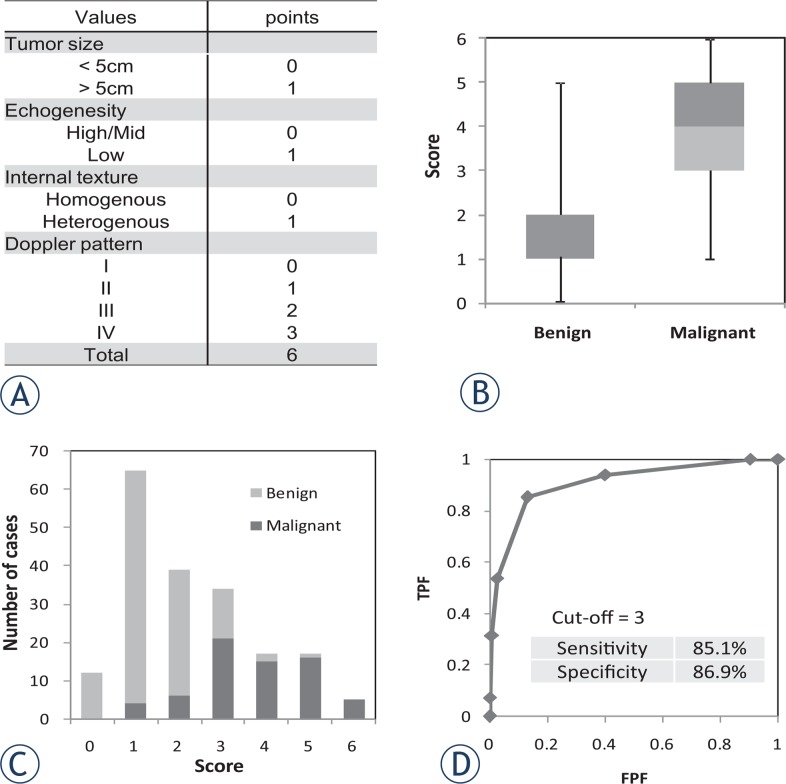
Evaluation of new scoring system for soft tissue tumours with ultrasound. **(A)** The new scoring system for differentiation between benign and malignant soft tissue tumours was established by combining four parameters (tumour size, echogenicity, internal structure, and Doppler pattern). The score was designated the ultrasound-based sarcoma screening (USS) score. **(B)** All cases in this study were analysed using the USS score. The average scores of benign and malignant tumours were 1.47 ± 0.90 and 3.71 ± 1.30, respectively. **(C)** Distribution of benign and malignant tumours for each score. As the score increased, the incidence of malignant tumours increased. **(D)** Receiver-operating curve analysis of the USS score revealed a cut-off value of 3 points. TPF = true-positive fraction; FPF = false-positive fraction

We re-evaluated all cases using the USS score, which comprised four values, to determine whether we could improve the diagnostic accuracy (Figure 2A). The mean scores in the benign and malignant groups were 1.47 ± 0.93 and 3.42 ± 1.30, respectively. Malignant tumours showed significantly higher USS scores than did benign tumours (p < 1×10^−10^) (Figure 2B). The ratio of malignant tumours to all cases at each USS score (0–6 points) was 0% at 0 points and 8% at 1 point; this increased to 93% and 100% at 5 and 6 points, respectively (Figure 2C). The area under the curve was 0.88 by ROC analysis, suggesting that USS scoring is diagnostically superior to the Doppler classification described by Giovagnorio *et al*.^13^ (Figure 2D). Based on the cut-off value (3 points) calculated by ROC curve analysis, the sensitivity and specificity for a diagnosis of malignant SPT was 85.1% and 86.9%, respectively.

Finally, we examined the average score of each pathological tumour type (Table 1,2). Among benign tumours, vascular tumours (haemangioma, angioleiomyoma, etc.) showed significantly higher scores than did all other benign lesions (p = 0.003) (Table 1). Among malignant tumours, well-differentiated liposarcoma (WDL) showed significantly lower scores than did all other malignant tumours (p = 0.0004) (Table 2). However, the score for WDL (2.15 ± 0.95) was significantly higher than that for lipomatous benign lesions (1.41 ± 0.91; p = 0.02).

## Discussion

**FIGURE 3.**
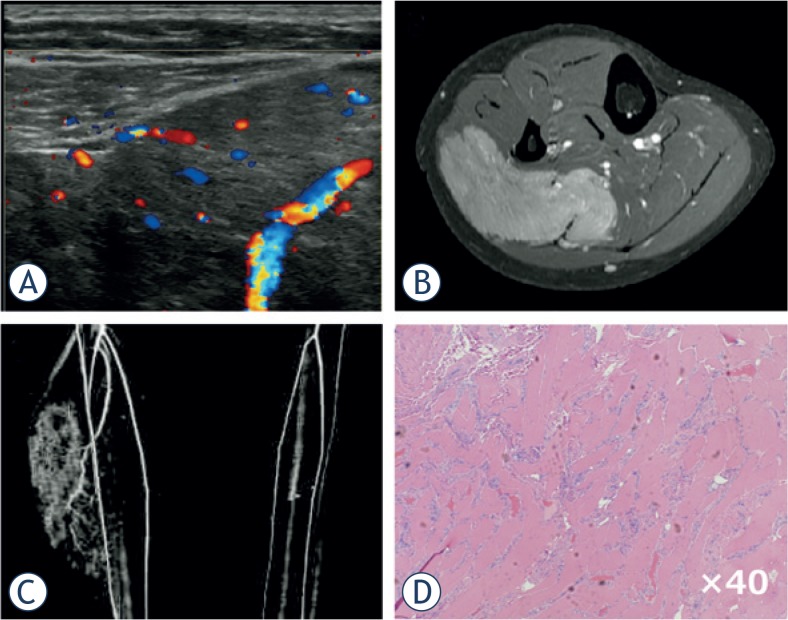
Intramuscular haemangioma of gastrocnemius. A 19-year-old woman presented with a swelling and mild pain in her right lower leg. She experienced increased swelling and pain after exercise or long walks. **(A)** Ultrasound revealed an ill-defined mass with a mixed inner texture in the calf muscle. Colour Doppler examination showed multiple vessels within the tumour, corresponding to type IV in the classification described by Giovagnorio et al. **(B)** MRI showed a mass with an irregular border in the soleus muscle. **(C)** CT angiography revealed multiple vessels branching from the tibial artery. **(D)** Pathological analysis of a biopsy specimen demonstrated multiple vessels between the skeletal muscles with no atypia.

**FIGURE 4.**
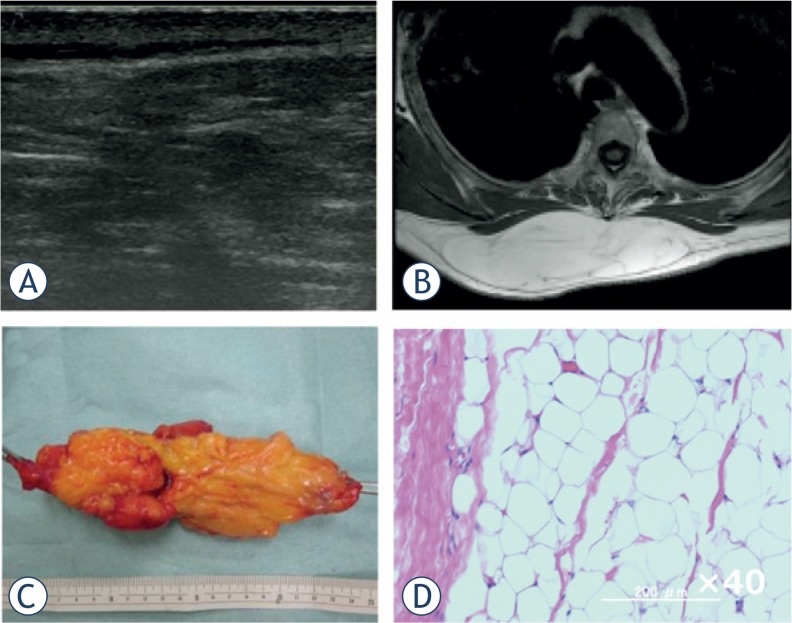
Subcutaneous lipoma of the back. A 55-year-old woman presented with a subcutaneous mass on her back. **(A)** Ultrasound revealed a highly echoic mass with a mixed inner texture and no intratumoural vessels by Doppler echo. **(B)** MRI showed a high-intensity mass within the subcutaneous fat tissue on T1-weighted images. **(C)** Gross appearance of the marginally resected tumour. **(D)** Pathological examination of the tumour revealed mature adipocytes with no atypical cells.

**FIGURE 5.**
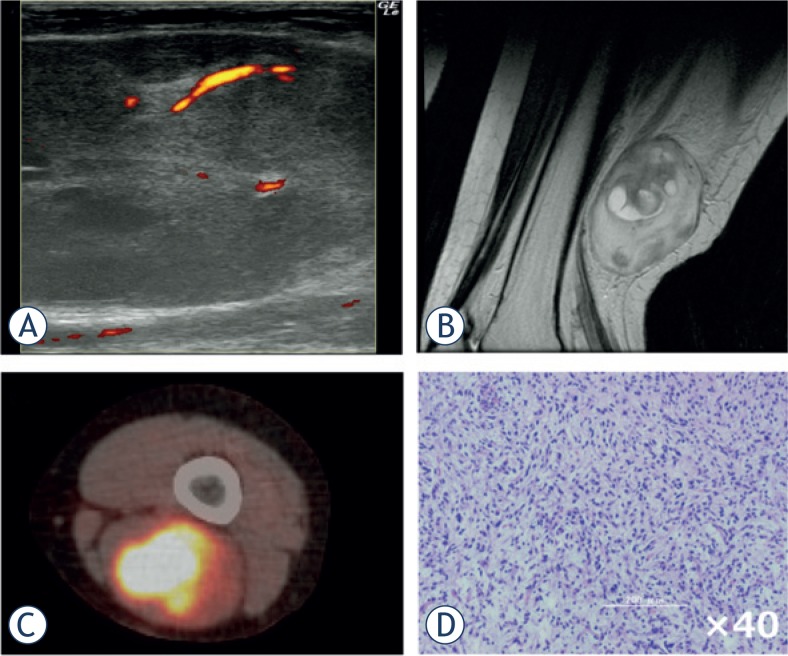
Myxofibrosarcoma of the thigh. A 52-year-old woman presented with a firm mass in her popliteal region. **(A)** Ultrasound imaging demonstrated a mixed-echoic mass with an irregular inner texture. Power Doppler revealed a relatively large vessel accompanying multiple small vessels (type IV). **(B)** MRI revealed a mass of both high and low intensity on T2-weighted imaging. **(C)** Positron-emission tomography with fluorodeoxyglucose isotope revealed a tumour with high accumulation and no distant metastases. **(D)** Pathological examination of the biopsy specimen demonstrated abundant atypical tumour cells with nuclear pleomorphism.

Advances in imaging technology have provided clinicians multiple diagnostic choices for each individual patient. Among several imaging modalities, ultrasound is the most feasible method with which to screen for SPTs if the evaluation method is established. Our analysis, which was based on a higher number and greater variety of cases than that by Giovagnorio *et al*.^13^, revealed that the specificity for malignant SPT was relatively good (0.91) by Doppler ultrasound alone, but that the sensitivity was poor (0.42). Therefore, we established a novel scoring system (USS score) that can be easily used in orthopaedic outpatient clinics and evaluated whether it can improve the diagnostic sensitivity and specificity without using special equipment. Generally, the probability of malignancy increases as the tumour diameter increases. Grimer^16^ reported that a diameter of 5 cm was a significant prognostic factor in patients with soft tissue sarcoma, and recommended that patients should undergo a medical examination if their mass is golf ball-sized or larger (≥ 4.2 cm). We therefore included the tumour size (cut-off of 5 cm) as a simple value incorporated into the USS score. Although a highly significant difference was shown in the USS score between the benign and malignant groups, there were several exceptions. We thus analysed benign cases with USS scores of ≥ 3 points. Among vascular tumours, 40% showed high USS scores (≥ 3), followed by peripheral nerve sheath tumours (PNSTs) (20.6%). This result suggests that these benign tumours require other factors or imaging studies for an accurate diagnosis. Fortunately, these two tumour types exhibit characteristic clinical presentations; *i.e*., changeable size and symptoms for vascular tumours (Figure 3) and a Tinel-like sign for PNSTs. On the other hand, WDL, known as a low-grade malignancy without metastatic potential^17^, exhibited a low USS score (≤ 2 points) in 9 of 13 cases (64.3%) in the present study. Preoperative differential diagnosis between benign lipomatous tumours and WDL is not easy, even with magnetic resonance imaging (MRI). Although the average USS score w as significantly higher in WDL than in benign lipomatous tumours, differential diagnosis between the two groups was not possible. As mentioned above, because WDL does not metastasise and rarely de-differentiates into high-grade liposarcoma, we and others treat such cases by marginal resection, as for benign lipomatous tumours (Figure 4).^17^ Adjuvant radiotherapy following re-section of WDL was recently shown to reduce local recurrence.^18^ Therefore, differentiation of a WDL from a benign tumour is not clinically critical, but differentiation between a high-grade sarcoma (Figure 5) and a benign tumour should be achieved with high accuracy.

**FIGURE 6.**
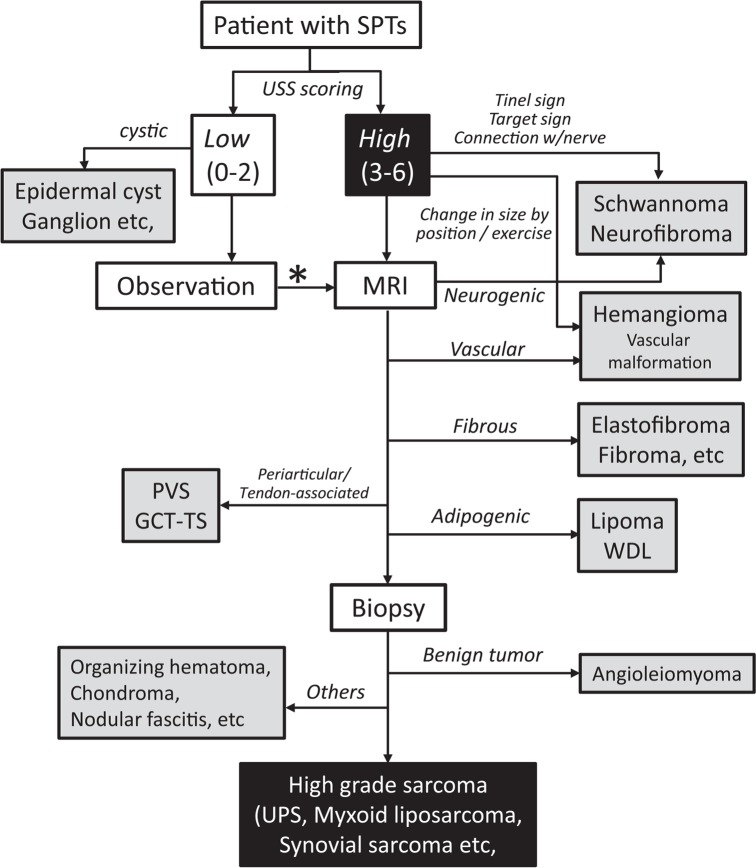
Flowchart of diagnosis of soft-part tumours based on ultrasound scoring. This flowchart proposes a screening procedure for soft-part tumours (SPTs). The screening procedure is mainly based on stratification by the ultrasound-based sarcoma screening (USS) score. The USS score is evaluated at the initial visit (0–6 points). If the score is low (0–2 points), the patient can be observed periodically over several months. If the score increases or the tumour size rapidly increases on the second visit or later, the physician may consider performing MRI (*). If the score is high at the initial visit (3–6 points), MRI may be recommended. In this group, peripheral nerve sheath tumours and vascular tumours may be diagnosed based on characteristic clinical symptoms without MRI. MRI can usually be used to diagnose typical fibrous tumours, lipomas, and pigmented villonodular synovitis (PVS) / giant cell tumour of the tendon sheath (GCT-TS). When malignant SPT is suspected, biopsy should be performed to obtain a pathological diagnosis. UPS = Undifferentiated pleomorphic sarcoma; WDL = Well-differentiated liposarcoma

The patient’s medical history and tumour-related symptoms should be routinely obtained by the physician. We recommend using the USS score in combination with such information to improve the differential diagnostic process. Figure 6 shows a flowchart of the diagnostic process of SPTs based on our analysis. The USS score was used to stratify the cases into high- and low-score groups, which mainly contained malignant and benign cases, respectively. Characteristic symptoms should be combined with ultrasound findings; this will lead to a diagnosis of vascular tumours or PNSTs. If the diagnosis is not conclusive, MRI should be performed. Among benign tumours, MRI can differentiate between fibrous tumours, pigmented villonodular synovitis and histologically related giant cell tumours of the tendon sheath, and lipomatous tumours. Other rare tumours or tumour-like lesions (nodular fasciitis, organising haematoma, or soft tissue chondroma) should be diagnosed by multiple modalities or resectional biopsy. Because the majority of tumours with high USS scores may be malignant, procedures should be chosen carefully for those cases. In the present study, 49 of 73 cases (67%) in the high USS score group were high-grade sarcomas. After careful preoperative assessment, we usually perform a biopsy to obtain a pathological diagnosis for possible cases of malignant SPT. Ultrasound-guided fine-needle aspiration is reportedly useful for soft tissue sarcoma.^19^,^20^ However, because of the shortage of specialised cytologists and the difficulty in obtaining sufficient material, we have not used this method. We are planning to start a prospective study to evaluate the efficacy of fine-needle aspiration as a diagnostic test for SPTs.

In conclusion, our USS score achieved high sensitivity as a screening test for SPT without compromising specificity. Preoperative diagnosis of most SPTs would be possible by combining the USS score with characteristic clinical symptoms and MRI findings.

## Figures and Tables

**TABLE 1.** USS score of benign tumors.

**Tumor type**	**Cases**	**Score (average)**	**95% CI**
PNST	34	1.59	0.36
lipoma et	29	1.41	0.33
Cystic lesion	20	1.20	0.23
PVS/GCT	10	1.60	0.43
Vascular tumor	10	2.30	0.78
Fibroma et	8	1.13	0.42
Other tumor / mass	11	1.09	0.43
Total	122	1.47	0.16

PNST = Peripheral nerve sheath tumor; PVS = Pigmented villonodular synovitis; GCT = Giant cell tumor

**TABLE 2.** USS score of malignant tumors

**Tumor type**	**Cases**	**Score (average)**	**95% CI**
UPS/MFH	23	3.87	0.45
Liposarcoma	15	3.13	0.58
WDL	14	2.15	0.5
Synovial sarcoma	3	4.67	0.65
Leiomyosarcoma	3	2.67	0.65
Other sarcoma	9	4.44	0.74
Total	67	3.42	0.31

PS = Unclassified pleomorphic sarcoma; MFH = Malignant fibrous histiocytoma; WDL = Well-differentiated liposarcoma
